# Goji berry (Lycium barbarum) inhibits the proliferation, adhesion, and migration of oral cancer cells by inhibiting the ERK, AKT, and CyclinD cell signaling pathways: an in-vitro study

**DOI:** 10.12688/f1000research.129250.3

**Published:** 2023-03-01

**Authors:** Amee Sanghavi, Ananth Srivatsa, Divya Adiga, Aditi Chopra, Richard Lobo, Shama Prasada Kabekkodu, Shivaprasada Gadag, Usha Nayak, Karthik Sivaraman, Ashmeet Shah

**Affiliations:** 1 Manipal College of Dental Sciences, Manipal, Manipal Academy of Higher Education, Manipal, Karnataka, 576104, India; 2 Manipal School of Life Sciences, Manipal Academy of Higher Education, Manipal, Karnataka, 576104, India; 3 Manipal College of Pharmaceutical Sciences, Manipal, Manipal Academy of Higher Education, Manipal, Karnataka, 576104, India

**Keywords:** Nutrition, Food, Fruit, Functional food, Traditional Chinese Medicine, Cancer, Oral Cancer, Goji Berry, Herbal; Lycium barbarum; Oral squamous cell carcinoma

## Abstract

**Background**: *Lycium barbarum (L. barbarum), *popularly referred to as Goji berry, is a promising herb known for its powerful anti-antioxidant, antibacterial, and anti-inflammatory properties. It is used in traditional Chinese medicine for treating inflammatory and infectious diseases. It has also shown good anti-cancer properties and has been tested against liver, colon, prostate, breast, and cervical cancers. However, no study has yet evaluated the role of goji berries against oral cancer. Hence, the present paper aims to evaluate the anticancer properties of *L. barbarum* against oral squamous cell carcinoma.

**Method**: Ethanolic extract of *L. barbarum* (EELB) was tested for its anticancer properties by performing the 3-(4,5-dimethylthiazol-2-yl)-2,5-diphenyl-2H-tetrazolium bromide (MTT) assay, colony formation, cell proliferation, and scratch wound test. The impact of EELB on the signaling transduction pathways of Extracellular signal-regulated kinase (ERK1/2), protein kinase (AKT1), cyclin D1 and epithelial-mesenchymal transition (EMT) was also assessed by western blot.

**Results: **The results showed that EELB can impede CAL-27 cell growth, proliferation and migration *in-vitro*. It even reduced the phosphorylation of ERK1/2 and AKT1 with concomitant downregulation of cyclin D1 (CCND1), cadherin 2 (CDH2), and vimentin (VIM) and upregulation of cadherin 1 (CDH1) expression suggesting its anti-proliferative and anti-EMT effects in oral cancer.

**Conclusion: **Goji berry has good antiproliferative and anti-invasive properties. It affects potential EMT markers and signaling transduction pathways involved in oral cancers. Hence goji berry can be tried as a potential anticancer agent to manage oral squamous cell carcinoma.

## 1. Introduction

Oral cancer is a malignant neoplasm commonly affecting the buccal mucosa, lip, gingiva, palate, tongue, and floor of the mouth. ^1^^,^^2^ It is considered the sixth most common cancer worldwide. ^1^ Among all the oral cancers, oral squamous cell carcinoma (OSCC) is the most common variant. ^3^^–^^5^ According to the World Health Organization (WHO), approximately 6,57,000 patients suffer from oral and throat cancer every year, of which more than 330,000 patients die annually. The mortality is higher among the central and south Asian countries. This is because of the high use of tobacco and areca nut/betel chewing. ^3^^,^^4^ Apart from tobacco, use of beedis and cigars, low socioeconomic status, high alcohol consumption, poor oral hygiene, poor diet, and increased incidence of Human Papilloma Virus (HPV), syphilis, and chronic candidiasis are some of the other risk factors that increase the prevalence of oral cancer in southeast Asian countries. ^6^^–^^8^

Oral cancers are managed by radiation, chemotherapy, and surgery. ^9^ However, these treatment options often deteriorate the quality of life and have a high risk of post-treatment morbidity. Patients on long-term chemotherapeutic agents often report chronic fatigue, hair loss, easy bruising, onset of infection, anemia, nausea, vomiting, loss of appetite, constipation, diarrhoea, renal dysfunction, changes in libido, heart problems, reduced lung capacity and difficulty in breathing, or bone and joint problems. ^10^ The relapse of cancer cells, development of resistance to chemotherapy, and toxic effects on the other healthy tissues have also been reported with extensive use of chemotherapy. Owing to these problems, researchers are constantly exploring newer alternatives to manage cancer.

Plant-derived phytochemicals and derivatives have gained tremendous popularity in managing various cancerous lesions, including oral cancer. ^11^ Several compounds derived from plants are being used for managing various types of cancer. The herbal medicine or phytomedicine market is likely to reach $550 billion by the end of 2030. ^11^^–^^17^ Natural products have been tried as adjuncts to conventional chemotherapeutic agents and have been “documented to decrease the lethal effect of using high doses of chemotherapeutic agents. This in turn improves the quality of life and overall survival rate of cancer patients”. ^16^ A study reported that around half (50%) of anticancer drugs that were approved from the year 1940 to 2014 were natural products or most of them were directly procured from plants. ^14^ Another study also reported that more than 60% of patients suffering from cancer used plant-based products instead of the conventional chemotherapeutic agent. ^6^ Talib *et al.* (2020) stated that “products derived from natural sources, such as plant-derived products are more accessible, less expensive and toxic compared to those produced synthetically”. Moreover, natural products have multiple compounds that act by varied mechanisms to inhibit the growth of cancer cells and prevent the onset of drug resistance. ^17^ Many recent studies have found that when natural compounds are used along with conventional chemotherapeutic agents and radiation therapy, they can sensitize the tumor cells and have a synergistic effect. ^11^^–^^18^ A combination therapy may enhance the therapeutic efficacy and reduce the required effective doses of chemotherapeutic agent. ^18^^–^^20^ Many plant-derived products like allicin, curcumin, cinnamon, saffron, reservatol, shagol, etc. have been tested for managing oral cancer. ^21^

Recently, *L.* *barbarum*, commonly known as goji berry; Chinese wolfberry; Himalayan goji, and Tibetan goji has emerged as a promising herb with anticancer, anti-inflammatory, and antioxidant properties. ^22^^–^^25^ However, its effect in managing oral cancer has not been reported previously. *L. barbarum* belongs to the family ‘Solanaceae’. The plant is native to southeast European and Asian countries. It is commonly used as a dry fruit in the Himalayan, China, and Tibetan regions. It is also used as a traditional Chinese medicine. ^22^^,^^24^ *L. barbarum* fruit contains abundant polysaccharides ( *L. barbarum* polysaccharides or (LBPs), scopoletin, vitamin C analog (glucopyranosyl, L, ascorbic acid analogs), βeta-carotene, zeaxanthin, and flavonoids that have promising antioxidant, immuno-modulating and anticancer properties. ^24^^–^^26^ Clinical and preclinical studies have confirmed the medicinal and therapeutic effects of *L. barbarum* for managing chronic fatigue, aging, stroke, ulcerative colitis, glaucoma, diabetes mellitus, and Alzheimer's disease. *L. barbarum* extract has shown promising results against cancers of the breast, liver, leukemia, colon, rectum, prostate, and cervix. ^21^^–^^31^ Mao *et al*. (2011) observed that around 200 to 1,000 mg/l of polysaccharide in goji berry, known as lycium barbarum polysaccharide (LBP) can inhibit the proliferation of the cancer of the colon by inducing G0/G1 arrest. ^29^ Shen and Du (2012) also confirmed that LBP has antiproliferative effects against breast cancer cells and can arrest the cell cycle at S-phase. ^30^ Although the antiproliferative and anticancer properties of *L. barbarum* are gaining popularity; to the best of our knowledge, studies evaluating the anticancer efficacy of *L. barbarum* against oral squamous cell carcinoma (OSCC) have not been reported. Hence, this paper aims to evaluate the anticancer effect of *L. barbarum* on the human OSCC cell line (CAL-27).

## 2. Methods

The study is an *in-vitro* analysis of anticancer properties using the CAL-27 cell lines (RRID: CVCL-1107, ATCC, USA). The study was conducted from 9 ^th^ July 2019 to 8 ^th^ July 2020. The study was done after obtaining the institutional ethics committee approval (IEC no: 460/2019). Goji berry ( *Lycium barbarum* L) was obtained from China's official distributor (Kenny delights Pvt. limited). Berry authentication was done by Gopal Krishna Bhat, a retired taxonomist of ‘Poornaprajna College’ at Udupi, Karnataka, India. The sample was authenticated and the berries were deposited at the ‘Manipal College of Pharmaceutical Sciences, Department of Pharmacognosy with voucher ref no: PP626.

### 2.1 Preparation of *L. barbarum* extract

**Figure 1.**
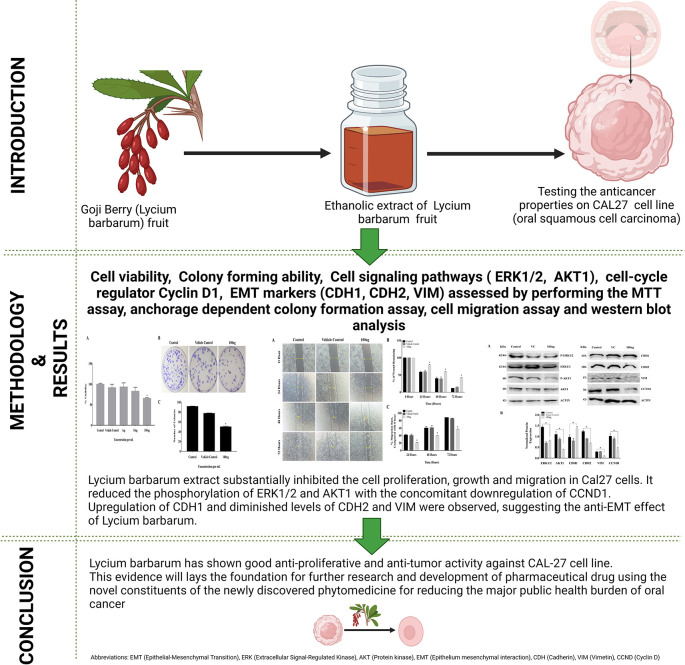
Schematic representation of the study design (Created in Biorender).

The fruits (berries) of *L. barbarum* were first thoroughly cleaned with distilled water and then dried using a hot air oven (at 45 ^o^C). The berries were then grounded and powdered using a crusher to obtain a dried powder. Around 250 minced powder of the berries was subsequently macerated with 1000 mL of ethanol solution for three days and they were occasionally shaken to allow the ethanol to mix with the powder. The macerated solution was then strained, and the prepared solvent was evaporated *via* a rota evaporator. The extract prepared was brown in color, sticky and semisolid in consistency, and had a fruity fragrance. The extract was collected and stored in a desiccator ^30^ ( Figure 1).

### 2.2. Test for anticancer properties of extract of *L. barbarum* (EELB)

2.2.1 Cell culture

The OSCC cell line (CAL-27, RRID: CVCL-1107, ATCC, USA) was used for all the experiments. The cells were cultured in Dulbecco's Modified Eagle Medium (DMEM) with 10% of fetal bovine serum (Himedia, India) at a temperature of 37°C in 5% CO _2_. The adherent cell monolayers were grown to 70%—80% confluency and then the cells were harvested using trypsin solution (0.25%, Himedia, India) before the experimentation. The EELB extract dissolved in dimethyl sulfoxide (DMSO) was used in all the experiments. The cells treated with DMSO (0.1%) were considered as vehicle control and the cell cultured in a complete medium without EELB extract served as control.

2.2.2 Cell viability assay

3-(4,5-Dimethylthiazol-2-yl)-2,5-Diphenyltetrazolium Bromide (MTT) assay was performed to determine the effect of ELLB on the growth and division of cancer cells. ^32^ The CAL-27 cells were first seeded at a concentration of around 5000 cells per well and were allowed to attach overnight. The cells were then treated with the extract at varying concentrations (1 μg, 10 μg, 100 μg per mL) for 48 hours. Cells treated with DMSO (0.1%) were considered as vehicle control. At the end of 48 hours, the cells were exposed for 4 hours to MTT (5mg/mL in phosphate buffer saline (PBS), Sigma Aldrich, USA). Following incubation with MTT, the media was aspirated, the formazan crystals were dissolved in DMSO solution, and the absorbance was measured at 570nm using a microplate reader (Varioskan, ThermoFischer Scientific, USA). The percentage of cell viability was calculated using the optical density and the background-corrected absorbance as follows: Percentage of cell viability = 100 X (Optical density of test group/Optical density of the control group). The data were represented as the mean ± SD of the percentage of cell viability.

2.2.3 Anchorage-dependent colony formation assay

The colony formation assay helps to check the viability of the cell based on the ability of a single cell to form colonies. ^1^ The colony-forming test is considered a gold standard assay to check the survival rate of cells upon the use of an anti-cancer agent. The experiment was performed using CAL-27 cells. ^31^ In brief, 100 cells were seeded in a 60mm cell culture petri dish and were exposed to 100 μg/mL of EELB. The media was replaced once in three days with a complete medium containing 100 μg/mL of EELB. The experiment was terminated at the end of 14 days. The cells which were treated with DMSO (0.1%) were used as vehicle control. At the end of 14 days, the media was discarded, and the cells were washed with PBS and stained for five minutes with 0.5% crystal violet dissolved in methanol. The excess stain was removed and cells were rinsed again with PBS. Following this, the cells were photographed using a gel documentation system. The experiment was performed in duplicates and repeated thrice.

2.2.4 Cell migration assay

Cell migration assay is used to measure the capability of a cancer cell to migrate, which is indirectly linked to its potency to invade the connective tissue and metastasize. The effect of EELB on cell migration of CAL-27 was evaluated by performing the wound healing assay. ^33^^,^^34^ CAL-27 cells were first seeded in a 6-well plate (1 × 10 ^5^ cells per well) and the cells were left to form a monolayer. A sterile microtip was used to make scratches in the cell monolayer. After making scratches, a fresh medium with EELB extract (100 μg/mL) was added to the test wells. The area of the wound remaining and the rate of migration of cells into the scratched area was examined for 72 hours. The scratched area was imaged at given time points with the help of a Rolera emc2 camera attached to an Olympus CK41 microscope (Olympus, Japan). The percentage cell migration rate and the percentage of wound remaining were calculated using the formulas; percentage migration rate = [(Area of the wound at 0 hour – Area of the wound at time T1)/Area of the wound at 0 hour] × 100, and the percentage of wound remaining = (Area of the wound at time T1/Area of the wound at 0 hour) × 100.

2.2.5 Western blot analysis

The results of the MTT assay, colony formation, and wound healing assay suggested the negative effect of EELB on CAL-27 cell proliferation, growth, and migratory ability. To evaluate the molecular alterations behind the observed effects, we further analysed the expression levels of the extracellular signal-regulated kinase (ERK1/2), Akt serine/threonine kinase1 (AKT1), cyclin D1 (CCND1), cadherin-1 (CDH1), cadherin-2 (CDH2) and vimentin (VIM). ERK1/2, AKT1 and CCND1 are well-known inducers of proliferation and survival in cancer cells. Loss of CDH1 and overexpression of CDH2 and VIM, which are linked to the epithelial-mesenchymal transition (EMT), are the frequent events during metastatic spread. The effect of EELB on these proteins would hint at the anticancer properties of *L. barbarum* against OSCC. The cells treated with DMSO (0.1%) were used as vehicle control and the cell cultured in complete medium without EELB extract served as a control. The western blot analysis was performed based on the previously described as follows. In brief, the proteins were extracted using RIPA buffer supplemented with protease inhibitor cocktail (Sigma Aldrich, USA), and the concentration of proteins was assessed using the Bradford assay kit (Sigma Aldrich, USA). Around 30 μg of the total protein was separated using 10% SDS PAGE, and transferred onto a Nitran membrane (BioRad, USA). The blots were blocked using 5% bovine serum albumin (BSA) (Himedia, India) for 1 hour and then probed separately with the primary antibodies [p-ERK1/2 (RRID:AB_2315112), ERK1/2 (RRID: AB_390779), p-AKT1 (RRID: AB_329825), AKT1 (RRID:AB_329827), CDH1 (RRID: AB_2291471), CCND1 (RRID: AB_2259616), CDH2 (RRID: AB_2687616), VIM (RRID: AB_10695459) and β-Actin (RRID: AB_330288); 1:5000, Cell Signalling Technology, USA)] overnight at 4°C. The phosphorylation site of AKT1 and ERK1/2 was Ser473 and Thr204/Tyr202, respectively. Following incubation with horseradish peroxidase (HRP) tagged secondary antibodies (1:5000; RRID: AB_2099233, Cell Signaling Technology, USA), blots were washed thrice (10mins each) with tris buffer saline containing 0.01% tween 20 (TBST) and visualized using enhanced chemiluminescence reagent (BioRad, USA). The images were taken using the ImageQuant LAS 4000 (GE Healthcare, USA). The antibody against β-Actin was used as a loading control. The band densities were quantified using ImageJ tool (RRID:SCR_003070, https://imagej.nih.gov/ij/). ^35^

2.2.6 Statistical analysis

All of the assays were conducted in duplicates and were repeated three times for reliability. The data in the bar graphs represent mean ± SD. A p-value less than 0.05 was considered statistically significant.

## 3. Results

### 3.1 EELB negatively regulates the colony-forming ability of CAL-27 and cell viability

**Figure 2.**
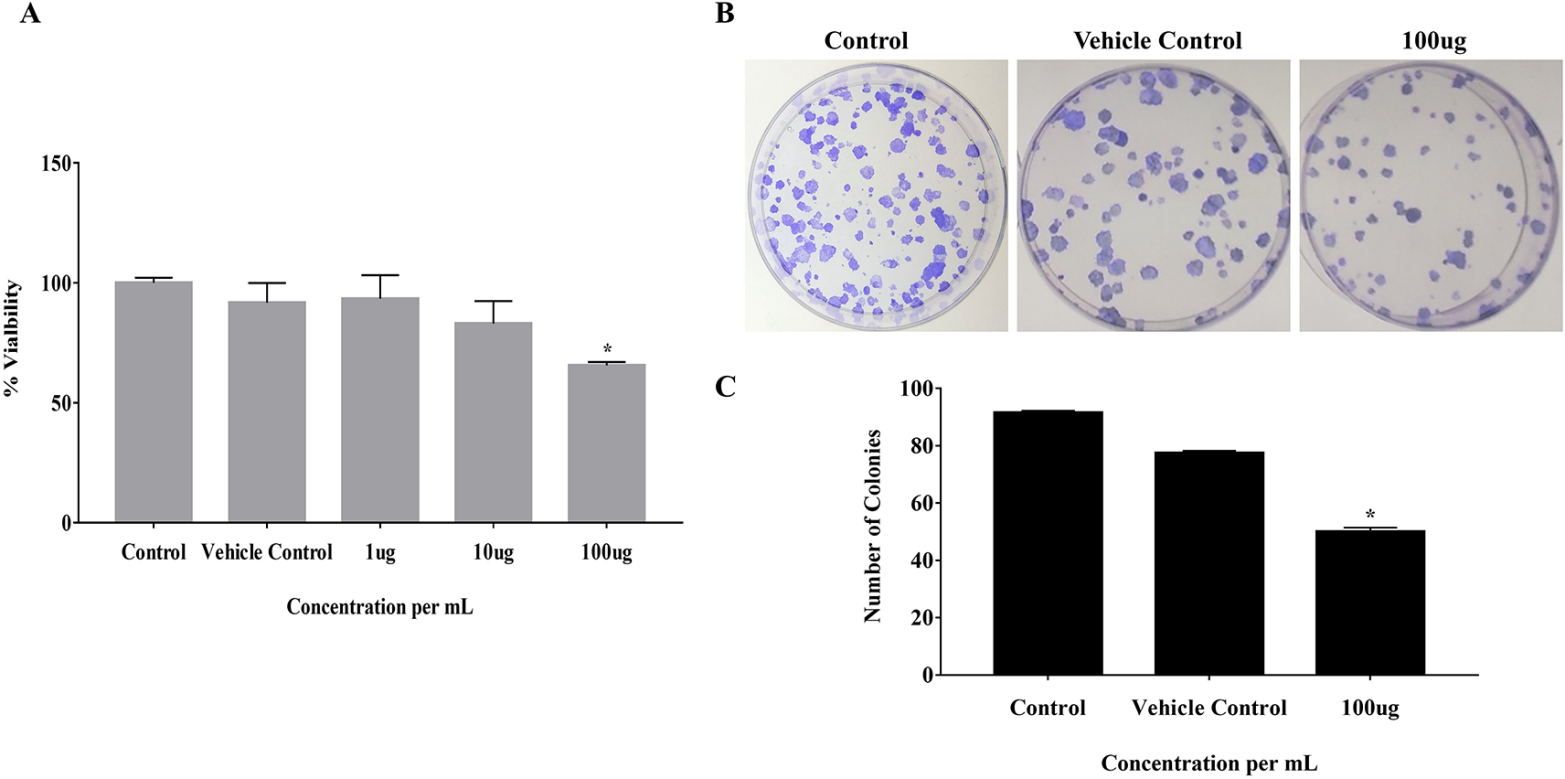
Effect of *L. barbarum* on cell viability and growth: (A) Cells treated with *L. barbarum* at different concentrations (1 μg, 10 μg, and 100 μg) for 24 hours. After incubation with MTT, absorbance was measured at 570nm and viability was calculated. Results are expressed as a percentage of viable cells. Data are expressed as mean ± SD values of three independent experiments, with P<0.05. A concentration- dependent decrease in the cell viability was observed when the CAL-27 cells were treated with different concentrations of *L. barbarum* for 24 hours. A concentration of 100 μg of *L. barbarum* extract inhibited cell proliferation with 65.5 % cell viability compared to the DMSO control (97.5%). (B) The effect of *L. barbarum* on the colony formation ability of CAL-27 cells was performed using clonogenic survival assays. Cells were pre-treated with different concentrations of *L. barbarum* (100 μg) for 24 hours. Cells treated with DMSO were kept as vehicle control and untreated cell with Dulbecco's Modified Eagle Medium (DMEM) media without DMSO were kept as control. *L. barbarum* effectively inhibited colony formation in CAL-27 cells. The colony number was significantly reduced upon treatment with the 100 μg of the extract. (C) Bar graph showing the reduced number of colonies after *L. barbarum* extract treatment. The experiment was performed in triplicate, with P-value <0.05 considered statistically significant.

The effect of EELB on the cell viability and colony-forming ability of cancerous cells was analyzed by MTT assay and anchorage-dependent colony formation assay, respectively. At 100 μg, EELB inhibited cell proliferation with 65.5% cell viability compared to 91.7% viability seen with the vehicle control group ( Figure 2A). EELB at 100 μg/mL significantly inhibited colony growth in CAL-27 cells ( Figure 1B, 1C). The number of colonies reduced significantly for the treated group compared to the vehicle control group. *L. barbarum* effectively inhibited colony formation in CAL-27 cancer cells. A reduction in the cancer cells’ vitality was observed when the CAL-27 cells were exposed to EELB.

### 3.2 EELB inhibits migration of oral cancer cells

**Figure 3.**
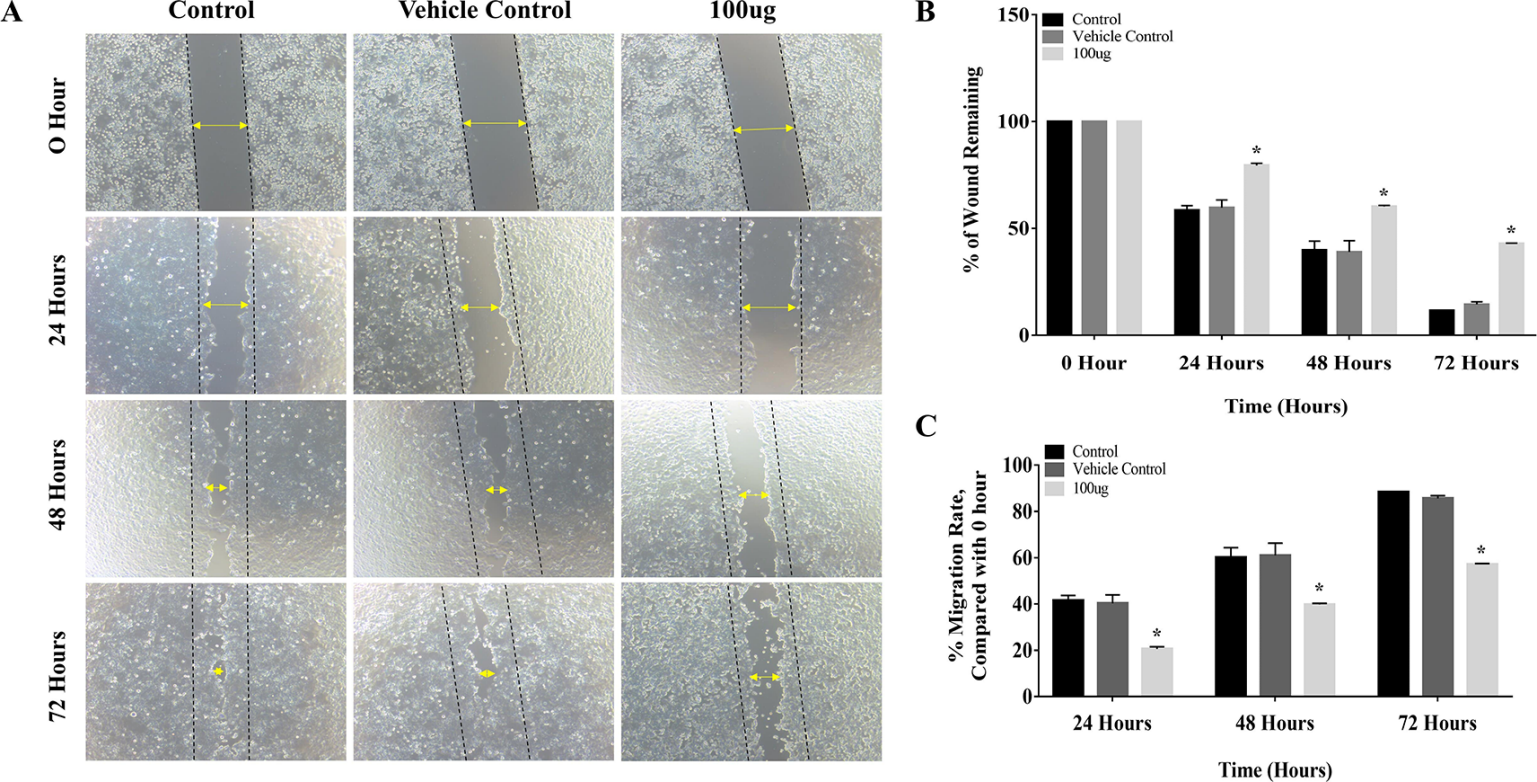
Inhibitory effect of *L. barbarum* extract on cell migration: (A) Treatment with *L. barbarum* significantly inhibited the migration of CAL-27 cells. At a concentration of 100 μg, a reduction in cell migration and an increase in the percentage of wound remaining were observed. At the end of 72 hours, the percentage of wounds remaining was 42.8% in the treated group whereas, in the untreated group, it was 11.5%. (B) and (C) Representative Bar graphs depicting the percentage of wounds remaining and percentage migration rate at the given time points.

The results of the cell migration assay proved that *L. barbarum* extract inhibited cell migration by decreasing the migration rate in CAL-27 cells. The distance between the edges of the wound was narrow at 24 hours, and it closed completely at 72 hours period in the control group ( Figure 3A). Treatment with *L. barbarum* lowered the migration of CAL-27 cells. At 72 hours, the percentage of the wound remaining for the control and the DMSO group was 11.5% and 14.3%, respectively ( Figure 3B). Nevertheless, at the end of 72 hours, *L. barbarum* extract was successful in inhibiting cell migration of CAL-27 cells. Approximately 42.8% of the wound remained at 72 hours in the treated group. Further, there was a significant reduction in the migration rate upon *L. barbarum* extract treatment (57.19%) as opposed to control cells (88.47%) ( Figure 3C). These results indicate the anti-migratory potential of *L. barbarum* against CAL-27 cells.

### 3.3 EELB controls the ERK1/2, AKT1-phosphorylation, and levels of EMT markers

**Figure 4.**
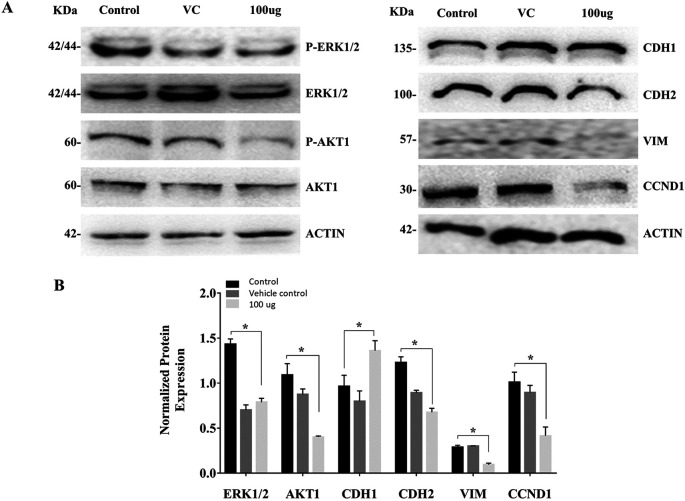
Effects of *L. barbarum* on ERK1/2, AKT1 phosphorylation, and EMT marker expression: (A) Representative western blot images showing reduced phosphorylation of ERK1/2 and AKT1 upon *L. barbarum* extract treatment. Higher CDH1 protein expression along with reduced levels of CDH2, VIM and CCND1 were observed when compared to the control in CAL-27 cells. (B) Bar graph showing normalized protein expression in treated and untreated groups. Densitometric analysis was performed using ImageJ software ( http://imagej.nih.gov/ij/) using β-actin as an internal control (VC). The intensities of each bands were normalized with the corresponding β-actin band intensities. The normalized protein expression for ERK1/2 and AKT1 was represented as the ratio of phospho-protein to total protein.

The cell treated with *L. barbarum* caused a reduction in the AKT1 and ERK1/2 phosphorylation. The reduction in AKT and ERK levels upon EELB treatment indicates its role in controlling cell proliferation and cell survival. Further, higher CDH1 protein expression along with reduced levels of CDH2, VIM, and CCND1 was noted in the group treated with EELB compared to the control group ( Figure 4A, 4B). Increased CDH1 and reduced CDH2 and VIM levels support the beneficial effect of ELLB in inhibiting cell migration and EMT in oral cancer cells.

## 4. Discussion

The present study assessed for the first time the anticancer potential of goji berry against OSCC. The results showed that goji berries extract can be tried for the treatment of oral cancer. The reduction in cell viability and effective inhibition of colony formation of cancer cells indicate that goji berry extract has antiproliferative properties. Additionally, EELB was found to reduce the migration of cancer cells, which in turn reflects its role in controlling the adhesion of cancer cells to the epithelial surface. EELB also affected the expression of ERK1/2, AKT1, CDH1, CDH2, VIM, and CCND1 proteins. This indicates its role in controlling the growth and migration of oral cancer cells. The anticancer properties of *L. barbarum* berry can be attributed to the constituents in the berry. *L. barbarum* berry contains abundant polysaccharides (LBPs), scopoletin, flavonoids, vitamin C analogs, carotenoids (β-carotene and zeaxanthin), β-sitosterol, cerebroside, betaine, amino acids, minerals, and vitamins (in particular, riboflavin and thiamin). Among all these constituents, the major active anticancer compounds in *L. barbarum* include scopoletin, LBPs, and 2-O-bD-glucopyranosyl-L-ascorbic acid. ^20^^–^^22^ These compounds are known for their anticancer, antioxidant, and immunomodulatory properties against important mediators for cell cycles such as ERK, Cyclin D, and AKT. ^20^^,^^27^^,^^29^

ERKs and AKT are some of the most crucial proteins that modulate cancer cell survival and proliferation. AKT helps in metastasis and is associated with the aggressiveness of the tumor. ^36^ AKT activation even inhibits the expression of proapoptotic proteins, such as BAD and BAX, and allows the cancer cells to survive. ^37^^,^^38^ AKT can also inactivate the caspase enzymes, which are directly involved in cell apoptosis and forkhead box protein O1 (FOXO-1) expression. This increases the risk of cell proliferation. AKT can upregulate cyclin D1, another critical mediator regulating cells to pass the G1 phase of the cell cycle and enter the S phase of the cell cycle. Our results found a reduction in ERK1/2, CCND1, and AKT1 expression, and these findings are important as they indicate a positive effect of EELB in controlling the proliferation and survival of cancer cells. ^35^^–^^38^ High expression of CDH1 could also be related to its role in tumor differentiation and inhibition of metastases. High CDH1 levels could increase cellular adhesion in epithelial tissues and reduce the risk of invasion from the epithelium to the connective tissue. A significant decrease in CDH2 and VIM in the group treated with EELB compared to the untreated and vehicle control group also supports the anti-EMT properties of EELB and its role in inhibiting the migration of CAL-27 cells. The regulation of cyclins and cadherins indicates that EELB could prevent the transition from the G1 to the S phase of the cell cycle and EMT.

These results are similar to previous studies where *L. barbarum* was found to modulate the levels of cyclins and CDKs such as CDK2, cyclin E, and CCND. ^25^ Activation of EMT signaling helps in cell invasion and migration. Besides, studies have reported that EMT activation can promote the migration of cells. ^34^^–^^38^ Previous studies have shown that *L. barbarum* can inhibit the cell cycle by regulating the expression of p53, p21, and BAX. ^23^ Zhang *et al*. and Luo *et al*. found that *L. barbarum* can break the strands of DNA and induce apoptosis with reduced BCL-2/BAX expression. ^35^^,^^36^^,^^38^^,^^39^ According to Cao *et al*. (1994), *L. barbarum* can even activate the macrophages and reduce lipid peroxidation, resulting in the death of cancer cells. ^36^ Additionally, LBPs can affect the natural-killer cells (NKs), enhancing the expression of Interferon-gamma and activate the receptor NKP30 on its surface. This increases the secretion of perforin and may induce the lysis of cancer cells. ^40^^–^^44^ Based on our results *in-vitro* settings and the existing evidence supporting the anticancer properties of *Lycium barbarum,* one should note that these results must be validated and confirmed by future clinical and patient-based studies.

## 5. Conclusion

*L. barbarum* has shown good anticancer properties in *in-vitro* settings. However, one should further explore its efficacy *via* clinical trials for treating oral cancer. Further studies can aim to evaluate and compare the role *of L. barbarum* compared to other anti-cancer agents. Additionally, future research at molecular and genomic levels can be undertaken to evaluate the other molecular mechanisms, signaling pathways, and genes by administrating *L. barbarum* in patients with OSCC *.* This evidence could help to develop novel therapeutic strategies for the management of one of the major public health and economic burdens, oral cancer.

### Ethical considerations

The study was conducted after receiving ethical approval from Kasturba Medical College and Kasturba Hospital Ethic committee with IEC no (460/2019).

## Data Availability

Figshare: [Raw data for effect of goji berry on CAL-27 cell lines (oral squamous cell carcinoma cell line]. https://doi.org/10.6084/m9.figshare.21716309. ^45^ The data is also available upon request *via* email to Dr. Aditi Chopra (email id: aditi.chopra@manipal.edu). Data are available under the terms of the Creative Commons Zero “No rights reserved” data waiver (CC0 1.0 Public domain dedication)
