# Supplementary material for: Goji berry (Lycium barbarum) inhibits the proliferation, adhesion, and migration of oral cancer cells by inhibiting the ERK, AKT, and CyclinD cell signaling pathways: an in-vitro study
Source: F1000Res. 2023 Mar 1;11:1563. Originally published 2022 Dec 22. [Version 3] doi: 10.12688/f1000research.129250.3 (PMC9887205; doi:10.12688/f1000research.129250.3)
Supplement: Peer review history

# Peer review: Reviewer response for version 2

**DOI:** 10.5256/f1000research.143864.r162575

**Published:** 2023-02-07

**Authors:** Swagat Ray

**Affiliations:** 1 University of Lincoln, Lincoln, UK

Unfortunately some of the amendments made as claimed by the authors in Version 2 seem to be still existing, e.g., MMT instead of MTT assay in Figure 1; vector control instead of vehicle control in Figure 4. 

 I would urge the authors to make the necessary changes to aid readability.

Is the work clearly and accurately presented and does it cite the current literature?

Yes

If applicable, is the statistical analysis and its interpretation appropriate?

Yes

Are all the source data underlying the results available to ensure full reproducibility?

Yes

Is the study design appropriate and is the work technically sound?

Yes

Are the conclusions drawn adequately supported by the results?

Yes

Are sufficient details of methods and analysis provided to allow replication by others?

Yes

Reviewer Expertise:

Cell and molecular biology; proteomics; genomics; genome stability.

I confirm that I have read this submission and believe that I have an appropriate level of expertise to confirm that it is of an acceptable scientific standard.

# Peer review: Reviewer response for version 1

**DOI:** 10.5256/f1000research.141919.r159541

**Published:** 2023-01-30

**Authors:** Poornima Rajendran

**Affiliations:** 1 Department of Periodontology, Sri Ramakrishna Dental College and Hospital, Coimbatore, Tamil Nadu, India

Many anti-oxidants have been tried and tested in the filed of dental medicine. The authors have used Goji berry in the present article. Though the methodology is quite similar to many other materials used so far, the authors have explained why goji berry can be employed to treat oral diseases. Especially their adequate discussion for their test results convinces the reader regarding the potential of the aforementioned agent. 

 Pros of the study:

1. Well explained methodology.
2. Discussion adequately justifies the results.

Is the work clearly and accurately presented and does it cite the current literature?

Yes

If applicable, is the statistical analysis and its interpretation appropriate?

Yes

Are all the source data underlying the results available to ensure full reproducibility?

Yes

Is the study design appropriate and is the work technically sound?

Yes

Are the conclusions drawn adequately supported by the results?

Yes

Are sufficient details of methods and analysis provided to allow replication by others?

Yes

Reviewer Expertise:

Periodontology and Implantology

I confirm that I have read this submission and believe that I have an appropriate level of expertise to confirm that it is of an acceptable scientific standard.

# Response

**Published:** 2023-01-31

**Authors:** Aditi Chopra

**Affiliations:** Manipal College of Dental Sciences, Manipal, India

Firstly we would like to thank the reviewers for spending their valuable time reading our work and understanding the importance of natural alternatives to treat oral disease, especially cancer, especially in current times where many medicine and therapy are becoming less effective or have many side effects. We have revised the manuscript according to the reviewers' comments and corrected the typo errors.

 Thank you.

# Peer review: Reviewer response for version 1

**DOI:** 10.5256/f1000research.141919.r159537

**Published:** 2023-01-30

**Authors:** Swagat Ray

**Affiliations:** 1 University of Lincoln, Lincoln, UK

The article discusses a study that aimed to evaluate the anticancer properties of the herb Lycium barbarum (commonly known as Goji berry) against oral squamous cell carcinoma. The study used an ethanolic extract of L. barbarum (EELB) and tested its effects on cancer cell growth, proliferation, and migration *in vitro*. The study found that EELB impeded the growth, proliferation and migration of oral cancer cells and reduced the phosphorylation of certain signaling proteins associated with cancer cell growth. The study also showed that EELB affected the expression of certain proteins involved in the process of epithelial-mesenchymal transition (EMT), a process that allows cancer cells to become more invasive.

 The results of the study suggest that Goji berry has good antiproliferative and anti-invasive properties against oral cancer cells. The study suggests that Goji berry could be tried as a potential anticancer agent to manage oral squamous cell carcinoma. However, it should be noted that this study was conducted in-vitro and further research is needed to confirm these findings in-vivo.

 Thorough proofreading is also recommended to avoid typos (e.g., 'MTT assay' is written as 'MMT assay' in Figure 1; Figure 4B legends - it should be 'vehicle control' instead of 'vector control').

Is the work clearly and accurately presented and does it cite the current literature?

Yes

If applicable, is the statistical analysis and its interpretation appropriate?

Yes

Are all the source data underlying the results available to ensure full reproducibility?

Yes

Is the study design appropriate and is the work technically sound?

Yes

Are the conclusions drawn adequately supported by the results?

Yes

Are sufficient details of methods and analysis provided to allow replication by others?

Yes

Reviewer Expertise:

Cell and molecular biology; proteomics; genomics; genome stability.

I confirm that I have read this submission and believe that I have an appropriate level of expertise to confirm that it is of an acceptable scientific standard.

# Response

**Published:** 2023-01-31

**Authors:** Aditi Chopra

**Affiliations:** Manipal College of Dental Sciences, Manipal, India

Firstly we would like to thank all the reviewers and the editors for taking the time out to review the paper. We are grateful for your valuable suggestions and comments.

 Responses to the comments are as follows: 

 1. comment 1: limitation of the study

**Response: **We have added the limitation of the present work at the end of the discussion and stated that the current work is done in-vitro settings and future studies should be done to explore the effect of goji berry in clinical settings.

 2. comment 2: Thorough proofreading is also recommended to avoid typos (e.g., 'MTT assay' is written as 'MMT assay' in Figure 1; Figure 4B legends - it should be 'vehicle control' instead of 'vector control').

**Response: **We apologize for the errors from our end. We have rectified the typo errors in the revised manuscript and in the figure section.

 Thank You.
